# Brain volumes and cortical thickness on MRI in the Finnish Geriatric Intervention Study to Prevent Cognitive Impairment and Disability (FINGER)

**DOI:** 10.1186/s13195-019-0506-z

**Published:** 2019-06-04

**Authors:** Ruth Stephen, Yawu Liu, Tiia Ngandu, Riitta Antikainen, Juha Hulkkonen, Juha Koikkalainen, Nina Kemppainen, Jyrki Lötjönen, Esko Levälahti, Riitta Parkkola, Pauliina Pippola, Juha Rinne, Timo Strandberg, Jaakko Tuomilehto, Ritva Vanninen, Miia Kivipelto, Hilkka Soininen, Alina Solomon, for the FINGER study group

**Affiliations:** 1 Department of Neurology, Institute of Clinical Medicine, University of Eastern Finland, Kuopio, Finland; 2 Department of Clinical Radiology, Kuopio University Hospital, Kuopio, Finland; 3 Public Health Promotion Unit, National Institute for Health and Welfare, Helsinki, Finland; 4 Division of Clinical Geriatrics, Center for Alzheimer Research, NVS, Karolinska Institutet, Stockholm, Sweden; 5 Center for Life Course Health Research/Geriatrics, University of Oulu, Oulu, Finland; 6 Medical Research Center Oulu, Oulu University Hospital and Oulu City Hospital, Oulu, Finland; 7 Combinostics, Tampere, Finland; 8 Turku University Hospital, Turku, Finland; 9 Turku PET Centre, University of Turku, Turku, Finland; 10 Seinäjoki City Hospital, Seinäjoki, Finland; 11 University of Helsinki, Clinicum, and Helsinki University Hospital, Helsinki, Finland; 12 Department of Public Health, University of Helsinki, Helsinki, Finland; 13 South Ostrobothnia Central Hospital, Seinäjoki, Finland; 14 Department of Neurosciences and Preventive Medicine, Danube-University Krems, Krems an der Donau, Austria; 15 Diabetes Research Group, King Abdulaziz University, Jeddah, Saudi Arabia; 16 Dasman Diabetes Institute, Dasman, Kuwait; 17 Ageing Epidemiology (AGE) Research Unit, School of Public Health, Imperial College London, London, UK; 18 Institute of Public Health and Clinical Nutrition, University of Eastern Finland, Kuopio, Finland

**Keywords:** Prevention, Cognitive impairment, Dementia, Randomized controlled trial, Lifestyle, Neuroimaging

## Abstract

**Background:**

The Finnish Geriatric Intervention Study to Prevent Cognitive Impairment and Disability (FINGER) was a multicenter randomized controlled trial that reported beneficial effects on cognition for a 2-year multimodal intervention (diet, exercise, cognitive training, vascular risk monitoring) versus control (general health advice). This study reports exploratory analyses of brain MRI measures.

**Methods:**

FINGER targeted 1260 older individuals from the general Finnish population. Participants were 60–77 years old, at increased risk for dementia but without dementia/substantial cognitive impairment. Brain MRI scans were available for 132 participants (68 intervention, 64 control) at baseline and 112 participants (59 intervention, 53 control) at 2 years. MRI measures included regional brain volumes, cortical thickness, and white matter lesion (WML) volume. Cognition was assessed at baseline and 1- and 2-year visits using a comprehensive neuropsychological test battery. We investigated the (1) differences between the intervention and control groups in change in MRI outcomes (FreeSurfer 5.3) and (2) post hoc sub-group analyses of intervention effects on cognition in participants with more versus less pronounced structural brain changes at baseline (mixed-effects regression models, Stata 12).

**Results:**

No significant differences between the intervention and control groups were found on the changes in MRI measures. Beneficial intervention effects on processing speed were more pronounced in individuals with higher baseline cortical thickness in Alzheimer’s disease signature areas (composite measure of entorhinal, inferior and middle temporal, and fusiform regions). The randomization group × time × cortical thickness interaction coefficient was 0.198 (*p* = 0.021). A similar trend was observed for higher hippocampal volume (group × time × hippocampus volume interaction coefficient 0.1149, *p* = 0.085).

**Conclusions:**

The FINGER MRI exploratory sub-study did not show significant differences between the intervention and control groups on changes in regional brain volumes, regional cortical thicknesses, or WML volume after 2 years in at-risk elderly without substantial impairment. The cognitive benefits on processing speed of the FINGER intervention may be more pronounced in individuals with fewer structural brain changes on MRI at baseline. This suggests that preventive strategies may be more effective if started early, before the occurrence of more pronounced structural brain changes.

**Trial registration:**

ClinicalTrials.gov, NCT01041989. Registered January 5, 2010.

## Background

The acute need for effective strategies to prevent dementia is increasingly emphasized [1]. Observational studies have pointed out many opportunities for prevention by addressing lifestyle, vascular, metabolic, and other modifiable risk factors [2, 3]. Clinical trials are now focusing more and more on early interventions in individuals at increased risk for dementia and/or in preclinical disease stages [1]. The hypothesis is that such individuals may benefit the most from preventive interventions since substantial, irreversible brain pathology has not yet occurred. The incorporation of biomarkers in lifestyle-based dementia prevention trials has also become increasingly important, both as trial outcomes and for assessing potential heterogeneity of intervention effects.

Many observational studies have linked modifiable lifestyle, vascular, or metabolic risk factors (individually and also multifactorial risk profiles) with structural brain changes relevant for cognitive decline and dementia, such as brain atrophy and white matter lesions (WML) [3–5]. However, the effects of lifestyle interventions on structural brain changes are still not fully clear. Only few lifestyle-based trials have so far included brain MRI markers. For example, randomized controlled trials assessing physical activity [6, 7], a multimodal social engagement program [8], or nutrition-related interventions [9, 10] have reported promising effects on various gray matter measures on MRI. These trials were conducted either in healthy older adults or in individuals who already had mild cognitive impairment (MCI) or prodromal Alzheimer’s disease. In another randomized controlled trial in hypertensive community-dwelling older individuals, multidomain vascular care did not seem to decrease WML progression [11].

The potential impact of pre-existing structural brain changes on the cognitive effects of lifestyle-based interventions also needs to be investigated. This is particularly important for gaining more insight into the window of opportunity for dementia prevention.

The Finnish Geriatric Intervention Study to Prevent Cognitive Impairment and Disability (FINGER) was the first large randomized controlled trial to report beneficial effects on cognition for a 2-year multidomain lifestyle intervention among older individuals with increased risk of dementia [12]. The FINGER trial protocol included an exploratory brain MRI sub-study [13]. This study presents exploratory analyses of intervention effects on changes in MRI measures (brain volumes, cortical thickness, and WML volume). The hypothesis was that the intervention may slow down atrophy and WML progression. In addition, we report post hoc sub-group analyses investigating the potential differences in the intervention effects on cognition between participants with more versus less pronounced structural brain changes. We hypothesized that individuals with less pronounced structural brain changes at baseline may have more cognitive benefit from the intervention.

## Methods

### Study population

The FINGER trial protocol [13], recruitment process [14], and primary findings [12] have been previously described in detail. In brief, the FINGER participants were 1260 individuals selected between September 7, 2009, and November 24, 2011, from previous population-based observational cohort studies [15–17]. The inclusion criteria were as follows: age 60–77 years; increased risk of dementia defined as ≥ 6 points on the Cardiovascular Risk Factors, Aging and Dementia (CAIDE) Dementia Risk Score [18]; and the Consortium to Establish a Registry for Alzheimer’s Disease (CERAD) neuropsychological battery [19] indicating cognitive performance at the mean level or slightly lower than expected for age according to the Finnish population norms [20]. Individuals with dementia, substantial cognitive impairment, and conditions affecting safe participation/cooperation, or concurrently participating in another trial were excluded.

The FINGER trial was approved by the Coordinating Ethics Committee of the Hospital District of Helsinki and Uusimaa, and all participants gave written informed consent at the screening and baseline visits. Participants in the FINGER MRI exploratory sub-study gave a separate consent for MRI scans.

**Fig. 1:**
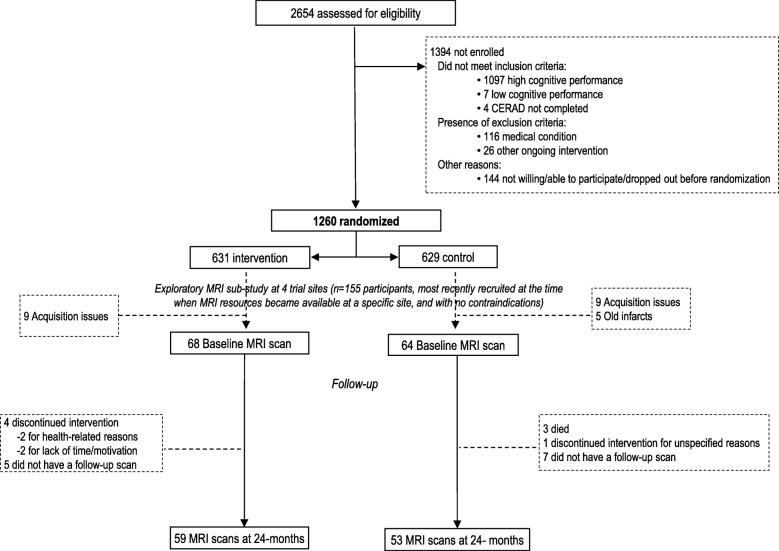
CONSORT diagram of the FINGER exploratory MRI sub-study. Consortium to Establish a Registry for Alzheimer’s Disease (CERAD)

The FINGER MRI exploratory sub-study included 155 participants from 4 trial sites. These participants were selected from the most recently recruited individuals when MRI resources became available at each site, and if there were no contraindications [5]. Brain scans were conducted in connection with the baseline FINGER visit (Fig. 1). The present study included 132 participants with baseline MRI scans, of which 112 had a repeat scan in connection with the 24-month FINGER visit.

### Randomization and intervention

Participants were randomly assigned to the intensive multidomain intervention group or regular health advice (i.e., control) group (1:1 ratio). Allocations were computer-generated in blocks of four (two individuals randomly allocated to each group) at each of the six study sites. Group allocation was not actively disclosed to participants, who were also advised not to discuss the intervention during the testing sessions. Outcome assessors were blinded to group allocation, and they were not involved in the intervention-related activities.

The multidomain intervention included four domains [13]. The *nutrition component*, based on the Finnish Nutrition Recommendations [21], included individual and group sessions supervised by study nutritionists. The *exercise component* followed international guidelines [22] and included gym sessions and aerobic exercise led by study physiotherapists [13]. *Cognitive training* was led by psychologists and included group sessions and computer-based individual training (web-based in-house developed program including tasks adapted from previous protocols) [23]. *Management of metabolic and vascular risk factors* followed national evidence-based guidelines [24–26]. The control group received regular health advice according to established guidelines.

### Cognitive outcomes

An extended version of the neuropsychological test battery (NTB) [27] was used for cognitive assessments at baseline and 12-month and 24-month visits. The primary trial outcome was change in the NTB total score, calculated as a composite score based on the results from 14 tests (*Z* scores standardized to the baseline mean and SD, with higher scores indicating better performance) [13]. Secondary outcomes included the following cognitive domains: an executive functioning score calculated from *Z* scores for category fluency test [19], digit span [28], concept shifting test (condition C) [29], trail making test (shifting score B–A) [30], and a shortened 40-stimulus version of the original Stroop test (interference score 3–2) [31]; a processing speed score calculated from *Z* scores for letter digit substitution test [32], concept shifting test (condition A), and Stroop test (condition 2); and a memory score calculated using *Z* scores for visual paired associates test immediate and delayed recall, logical memory immediate and delayed recall, and word list learning and delayed recall [19, 28].

### MRI assessments

Before quantitative analysis, an experienced neuroradiologist visually inspected the T1WI and FLAIR images. Images were excluded if there were brain lesions potentially affecting volumetry and/or scanning issues such as no full-brain coverage, artifacts, intensity inhomogeneity, and adequate gray/white matter contrast. One hundred thirty-two scans from 3 study sites passed quality control (all 18 scans from 1 site excluded due to acquisition issues, and 5 additional scans excluded due to old brain infarcts which may have affected the automated image analysis). Of the 132 participants, 112 were re-scanned in connection with the 24-month visit, and all scans passed quality control (Fig. 1). Regular phantom scans were performed, and quantitative measures of signal-to-noise ratio, uniformity, and geometric distortion were carried out at each site. The following MR systems were used: 1.5T Avanto, Siemens at the Kuopio and Oulu sites (3D-MPRAGE sequence, voxel size 1.2 × 1.2 × 1.2 mm^3^, TR 2400 ms, TE 3.5 ms, TI 1000 ms), and 3T Ingenuity, Philips at the Turku site (3D TFE sequence, voxel size 1.0 × 1.0 × 1.0 mm^3^, TR 8.1 ms, TE 3.7 ms). The same imaging parameters and MRI scanners were used for both baseline and 2-year scans at each site.

Regional brain volumes and cortical thicknesses were measured using FreeSurfer (version 5.3, http://surfer.nmr.mgh.harvard.edu/). If geometric inaccuracy in boundaries between white, gray matter, and CSF was present in the automated WM segmentation, then manual editing was conducted. FreeSurfer morphometric procedures have been demonstrated to show good test-retest reliability across scanner manufacturers and across field strengths [33, 34]. Brain volumes were normalized by the total intracranial volume (TIV) to account for between-person variations in head size [35].

WML volume was measured through the segmentation of WM hyperintensities according to a previously described method [36]. The method is based on the expectation–maximization (EM) algorithm, and the segmentation was done in three steps: (1) segment WM in two classes from T1 images, representing hypointense WM regions and normal bright WM regions; (2) using the results of the previous step as an initialization, segment the FLAIR images to three classes: CSF, normal brain tissue, and hyperintense voxels; and (3) using the results of the previous step as an initialization, segment the WM and subcortical regions from the FLAIR images in two classes. The class with higher intensities was then regarded as the segmentation of WM hyperintensities [36, 37].

### Statistical analysis

The baseline characteristics of the intervention and control groups in the FINGER MRI exploratory sub-study were compared using *t* test or chi-square test as appropriate. Analyses were done using Stata software version 12 (Stata Statistical Software: Release 12. College Station, TX: StataCorp LP). The level of statistical significance was *p* < 0.05 in all analyses.

#### Intervention effects on changes in MRI measures (regional brain volumes, cortical thickness, and WML volume)

Analyses included the 112 participants with repeat MRI scans. To extract reliable volume and thickness estimates for longitudinal analysis, these images were automatically processed with the longitudinal stream [34] in FreeSurfer. Differences between the intervention and control groups in change in MRI outcomes were investigated using FreeSurfer, and false discovery rate (FDR) correction for multiple comparisons was applied.

#### Sub-group analyses of intervention effects on cognition in participants with more versus less pronounced structural brain changes at baseline

The analyses included the 132 participants with baseline MRI scans. The following 4 MRI measures were considered: total gray matter (GM) volume, hippocampus volume, and WML volume (normalized to TIV), and a measure of cortical thickness in AD signature regions calculated as the average of cortical thickness in entorhinal, inferior temporal, middle temporal, and fusiform regions as previously described [38].

Zero-skewness log transformation was applied to skewed NTB components. *Z* scores for tests at each time point were standardized to the baseline mean and standard deviation. NTB total score and domain scores for executive functioning, processing speed, and memory were obtained by averaging individual NTB component *Z* scores. The minimum number of necessary NTB components was set to 8/14 for calculating NTB total score, 3/5 for executive functioning, 2/3 for processing speed, and 3/6 for memory.

Considering within-person and between-person variability over time, mixed-effects regression models (*xtmixed* command in Stata) with maximum likelihood estimation were used to analyze the change in cognitive scores as a function of randomization group (intervention versus control), time (years), MRI measure (above versus below the median), and their interactions (randomization group × time, group × MRI, time × MRI, and group × time × MRI) as fixed effects. Random effects of the models were variances and covariance of individual-level intercept and slope. We chose to define the MRI sub-groups based on the median value of baseline measures due to the lack of established pathologic cutoffs, especially for at-risk general populations such as FINGER participants.

We report the coefficient (95% CI) for the randomization group × time × MRI interaction as the main result, i.e., estimated difference in intervention effects per year between the MRI < median and MRI ≥ median groups. We also present the effect estimates (95% CI) within each MRI group (the randomization group × time interaction) using the *lincom* post-estimation command after *xtmixed* in Stata. All analyses were adjusted for the study site. Other covariates were considered only if they were significantly different between the intervention and control groups at baseline.

## Results

Characteristics of FINGER participants with and without MRI data at the three study sites where brain scans were available have been previously described in detail [5]. The MRI population was not significantly different in demographic, clinical, and cognitive characteristics from the population without MRI at these sites [5]. The intervention and control groups in the FINGER exploratory MRI sub-study were not significantly different in baseline demographic, clinical, cognitive, and MRI characteristics (Table 1).

**Table 1:** Characteristics of the intervention and control groups (participants with baseline MRI measurements)

Characteristics at baseline	Total, *n*	Intervention, *n* = 68	Control, *n* = 64	*p*
Demographic characteristics				
Age at the baseline visit (years)	132	70.3	69.8	0.50
Sex (women, %)	132	29 (42.6)	33 (51.5)	0.30
Education (years)	132	9.3 (2.9)	9.2 (2.6)	0.87
Baseline vascular factors				
Systolic blood pressure (mmHg)	129	139.6 (15.9)	139.0 (14.7)	0.83
Diastolic blood pressure (mmHg)	129	79.4 (8.9)	78.6 (8.9)	0.61
Fasting plasma glucose (mmol/l)	132	6.1 (0.77)	6.1 (1.03)	0.94
Body mass index (kg/m^2^)	129	27.9 (3.7)	26.9 (3.4)	0.12
History of hypertension (%)	128	46 (69.7)	36 (58.1)	0.17
History of diabetes (%)	128	7 (10.6)	8 (12.9)	0.68
Baseline lifestyle factors				
Physical activity 2 or more times/week (%)	128	50 (73.5)	48 (80.0)	0.38
Current smokers (%)	130	1 (1.49)	4 (6.3)	0.15
Alcohol drinking at least once/week (%)	131	31 (45.5)	30 (47.6)	0.81
Fish intake at least twice/week (%)	130	42 (61.8)	33 (53.2)	0.32
Daily intake of vegetables (%)	132	47 (69.1)	41 (64.0)	0.53
*Baseline MRI measures				
Total hippocampal volume, ml	132	7.4 (4.8–9.3)	7.2 (4.7–8.7)	0.18
Total intracranial volume, ml	132	1572.9 (1108.5–2032.7)	1545.4 (957.9–1955.9)	0.42
AD signature cortical thickness, mm	132	2.8 (2.4–3.1)	2.7 (2.4–3.1)	0.61
Total GM volume, ml	132	563.3 (450.7–669.7)	559.1 (420.7–695.8)	0.62
WML volume, ml	118	10.9 (12.5)	10.9 (14.3)	0.97
Cognitive measures				
Baseline				
NTB total score	132	− 0.12 (0.49)	− 0.009 (0.54)	0.22
Executive functioning	132	− 0.02 (0.55)	− 0.05 (0.60)	0.75
Processing speed	132	− 0.15 (0.80)	0.07 (0.75)	0.10
Memory	132	− 0.18 (0.58)	− 0.01 (0.60)	0.09
1 year follow-up				
NTB total score	130	0.04 (0.61)	0.05 (0.58)	0.95
Executive functioning	129	0.05 (0.61)	− 0.05 (0.68)	0.33
Processing speed	130	− 0.04 (0.86)	0.07 (0.76)	0.39
Memory	130	0.09 (0.76)	0.11 (0.68)	0.87
2 year follow-up				
NTB total score	122	0.09 (0.67)	0.09 (0.66)	0.98
Executive functioning	122	0.08 (0.65)	− 0.006 (0.70)	0.47
Processing speed	122	− 0.014 (0.94)	0.05 (0.85)	0.67
Memory	122	0.17 (0.77)	0.19 (0.78)	0.88

Values are means (SD) unless otherwise specified. Differences between the intervention and control groups were analyzed with chi-square and *t* tests as appropriate. Scores on the NTB total score, executive functioning, processing speed, memory, and abbreviated memory are mean values of *Z* scores of the cognitive tests included in each cognitive outcome. Higher scores indicate better performance. AD signature cortical thickness: cortical thickness in AD signature regions calculated as the average of cortical thickness in entorhinal, inferior temporal, middle temporal and fusiform regions as previously described [38]

*GM* gray matter, *WML* white matter lesions, *NTB* neuropsychological test battery

*MRI values are mean (minimum–maximum). MRI measures are based on longitudinal FreeSurfer analyses

Changes in MRI outcomes (regional brain volumes, regional cortical thicknesses, and WML volume) were not significantly different between the intervention and control groups (Table 2).

**Table 2:** MRI measures at baseline, follow-up, and annual rate of change

MRI measures	Total, *n*	Intervention, *n* = 59	Control, *n* = 53	*p*
Total intracranial volume, ml	112	1581.4 (1112.4–2039.1)	1524.4 (975.5–1962.1)	0.13
Baseline				
Total hippocampal volume, ml	112	7.2 (4.6–9.1)	7.0 (4.5–8.3)	0.33
AD signature cortical thickness, mm	112	2.8 (2.5–3.0)	2.8 (2.5–3.1)	0.87
Total GM volume, ml	112	576.7 (443.4–677.3)	563.4 (406.3–709.6)	0.18
WML volume, ml	100	11.9 (0.5–60.7)	11.7 (0.7–74.4)	0.95
2-year follow-up				
Total hippocampal volume, ml	112	7.0 (4.3–9.1)	6.8 (4.1–8.3)	0.27
AD signature cortical thickness, mm	112	2.7 (2.5–3.1)	2.7 (2.3–3.1)	0.47
Total GM volume, ml	112	568.6 (434.4–670.4)	556.0 (414.8–698.5)	0.19
WML volume, ml	100	13.6 (0.4–59.9)	13.0 (0.5–84.9)	0.86
*Annual rate of change, % (SD)				
Total hippocampal volume	112	− 1.30 (1.3)	− 1.50 (2.0)	0.56
AD signature cortical thickness	112	− 0.68 (0.98)	− 0.39 (1.2)	0.24
Total GM volume	112	− 0.70 (0.89)	− 0.60 (1.4)	0.64
WML volume	96	7.0 (28.4)	11.6 (50.3)	0.58

MRI values are mean (minimum–maximum). All MRI measures are based on longitudinal FreeSurfer analyses. AD signature cortical thickness: cortical thickness in AD signature regions calculated as the average of cortical thickness in entorhinal, inferior temporal, middle temporal, and fusiform regions as previously described [38]

*GM* gray matter, *WML* white matter lesions

*Annual rate of change was calculated as (24-month value − baseline value)/(baseline value × time)

The impact of baseline MRI measures on changes in cognition during the trial is shown in Table 3. The randomization group × time × AD signature cortical thickness interaction was significant for processing speed (*p* = 0.021), indicating that participants with higher baseline cortical thickness had more intervention benefit on processing speed compared with participants with lower cortical thickness. A similar non-significant trend was observed for hippocampal volume (*p* = 0.085). No other significant randomization group × time × MRI interactions were found.

**Table 3:** Intervention effects on cognition—sub-group analyses by baseline MRI measures

		Difference between the intervention and control groups per year (randomization group × time)		Difference between MRI > median and MRI < median (randomization group × time × MRI)	
		Estimate (95% CI)	*p*	Estimate (95% CI)	*p*
NTB total score (primary outcome)					
*Hippocampal volume	>median	0.056 (− 0.034–0.146)	0.225	0.030 (− 0.100–0.156)	0.668
	<median	0.028 (− 0.063–0.119)	0.551		
AD signature cortical thickness	>median	0.092 (0.002–0.181)	**0.044**	0.097 (− 0.029–0.222)	0.131
	<median	− 0.005 (− 0.093–0.083)	0.915		
*Total GM volume	>median	0.018 (− 0.072–0.108)	0.701	− 0.055 (− 0.184–0.073)	0.397
	<median	0.073 (− 0.020–0.160)	0.117		
*WML volume	>median	0.025 (− 0.063–0.113)	0.576	− 0.053 (− 0.178–0.073)	0.411
	<median	0.078 (− 0.012–0.170)	*0.088*		
Processing speed (secondary outcome)					
*Hippocampal volume	>median	0.144 (0.024–0.263)	**0.018**	0.149 (− 0.020–0.320)	*0.085*
	<median	− 0.006 (− 0.130–0.115)	0.927		
AD signature cortical thickness	>median	0.170 (0.050–0.290)	**0.005**	0.198 (0.030–0.365)	**0.021**
	<median	− 0.030 (− 0.146–0.090)	0.641		
*Total GM volume	>median	0.075 (− 0.044–0.194)	0.217	− 0.002 (− 0.172–0.170)	0.980
	<median	0.077 (− 0.044–0.198)	0.212		
*WML volume	>median	0.054 (− 0.070–0.174)	0.379	− 0.110 (− 0.280–0.060)	0.208
	<median	0.164 (0.042–0.290)	**0.009**		
Memory (secondary outcome)					
*Hippocampal volume	>median	0.040 (− 0.107–0.190)	0.586	− 0.046 (− 0.257–0.166)	0.671
	<median	0.087 (− 0.063–0.237)	0.256		
AD signature cortical thickness	>median	0.086 (− 0.062–0.234)	0.255	0.040 (− 0.168–0.250)	0.706
	<median	0.046 (− 0.100–0.192)	0.539		
*Total GM volume	>median	0.010 (− 0.140–0.160)	0.891	− 0.113 (− 0.325–0.100)	0.297
	<median	0.123 (− 0.028–0.270)	0.110		
*WML volume	>median	0.025 (− 0.130–0.179)	0.749	− 0.030 (− 0.248–0.190)	0.795
	<median	0.054 (− 0.102–0.210)	0.497		
Executive functioning (secondary outcome)					
*Hippocampal volume	>median	0.028 (− 0.073–0.128)	0.591	0.026 (− 0.117–0.170)	0.717
	<median	0.001 (− 0.100–0.103)	0.982		
AD signature cortical thickness	>median	0.070 (− 0.035–0.169)	0.197	0.100 (− 0.042–0.240)	0.169
	<median	− 0.033 (− 0.134–0.070)	0.515		
*Total GM volume	>median	− 0.006 (− 0.107–0.095)	0.909	− 0.048 (− 0.190–0.096)	0.514
	<median	0.042 (− 0.060–0.144)	0.422		
*WML volume	>median	0.021 (− 0.085–0.128)	0.693	− 0.048(− 0.200–0.105)	0.540
	<median	0.069 (− 0.039–0.178)	0.213		

AD signature cortical thickness: cortical thickness in AD signature regions calculated as the average of cortical thickness in entorhinal, inferior temporal, middle temporal, and fusiform regions as previously described [38]

*NTB* neuropsychological test battery, *GM* gray matter, *WML* white matter lesions

*For all volumetric measures, medians of TIV-normalized values were used

Values in bold represent *p*-value < 0.05; values in italics represent *p*-value < 0.10

Significant cognitive benefits (randomization group × time interaction) were found on NTB total score among participants with higher baseline cortical thickness and on processing speed among participants with higher hippocampal volume, higher cortical thickness, and lower WML volume at baseline. The differences in cognitive outcomes between the intervention and control groups were not statistically significant in participants with thinner cortex, lower hippocampal volume, or higher WML volume at baseline (Table 3).

## Discussion

In the FINGER MRI exploratory sub-study, no significant differences between the intervention and control groups were found on the changes in regional brain volumes, cortical thickness, or WML volume during the 2-year trial. However, post hoc analyses suggested that beneficial intervention effects on processing speed were more pronounced in participants with higher baseline cortical thickness in AD signature areas. A similar trend was observed in participants with higher baseline hippocampal volume. Within-group findings by baseline MRI measures also suggested a pattern of cognitive benefits particularly in participants with less pronounced structural brain changes (higher AD signature cortical thickness, higher hippocampal volume, and lower WML volume).

The FINGER trial was designed in a public health context, i.e., it targeted the at-risk segment of the general elderly population (not patients in a clinical setting). The intervention was started early, before the onset of dementia or substantial cognitive impairment [14]. This was the first prevention trial to select participants using a validated dementia risk score based on several modifiable risk factors [18]. Overall, structural brain changes in this at-risk population were not very pronounced during 2 years. For example, the annual rate of hippocampal atrophy was only slightly higher than previously reported for healthy older individuals [39]. This may have contributed to the lack of significant differences in MRI changes between the intervention and control groups.

In addition, the FINGER multidomain intervention addressed several risk factors simultaneously. A key principle was that multiple lifestyle changes (even of smaller magnitude) over a longer period of time would lead to longer-term benefits. While the intervention had significant beneficial effects on cognition in the entire trial population after 2 years [12], this interval may not have been enough to see significant effects on structural brain changes, at least not with the standard imaging methods used in this study. The ongoing 7-year FINGER extended follow-up will provide additional data on longer-term changes in brain MRI measures, as well as incident cognitive impairment and dementia.

In the present study, post hoc analyses suggested that intervention benefits on cognition (processing speed) were more pronounced when cortical thickness in AD signature areas and hippocampus volume were higher at baseline. Lower cortical thickness and hippocampus volume have been associated with poorer cognitive performance even in cognitively normal older individuals [40]. It is possible that more favorable brain MRI measure pre-intervention may indicate higher prevention potential, thus emphasizing the importance of starting preventive strategies as early as possible, before substantial brain changes and cognitive impairment have already occurred.

Post hoc findings in a trial sub-sample need to be interpreted very cautiously [41]. The FINGER trial has several pre-specified sub-group analyses [13], and in addition, the present post hoc results for four MRI measures and four cognitive outcomes were not corrected for multiple testing. Thus, while results suggest that starting prevention earlier may be associated with beneficial effects, no claims can be made about exactly how much cognitive benefit the intervention would provide below or above specific cutoffs for specific brain MRI measures. While MRI measures are related to cognitive performance, other factors such as cognitive reserve [42] may affect the overall cognition level and response to lifestyle interventions. Whether the window of opportunity for prevention closes at some point, and the potential combination of individual characteristics that may mark such a point, remains to be determined.

The main strengths of this study are the randomized controlled design with a multidomain intervention, longer duration than most previous cognition-focused lifestyle trials, and availability of MRI scans at both baseline and 24-month visits. The main limitation of the FINGER MRI exploratory sub-study is the relatively small sample size, which limited the statistical power and thus the ability to detect significant intervention effects on MRI measures, as well as tests of interaction in sub-group analyses of cognitive changes by baseline MRI measures. MRI scanners differed between sites, but this was adjusted for in all analyses, and the FreeSurfer morphometric procedures have shown good test-retest reliability across scanner manufacturers and field strengths [33, 34]. Although repeated cognitive testing may have led to practice effects in all participants, focusing on the differences in cognitive change between the intervention and control groups, and on how such differences were impacted by baseline MRI measures, most likely suggested cognitive benefits beyond practice effects.

## Conclusions

The FINGER MRI exploratory sub-study did not show significant differences between the intervention and control groups on changes in regional brain volumes, regional cortical thicknesses, or WML volume. Post hoc sub-group analyses of cognitive intervention benefits by more versus less pronounced structural brain changes at baseline suggested that strategies to prevent cognitive decline may be more effective if started early, before the occurrence of substantial structural brain changes.

Prevention trials with longitudinal MRI assessments and larger neuroimaging sample sizes are needed to further investigate the effects of healthy lifestyle management on brain structure, the impact of pre-existing brain changes on cognitive benefits, and whether a window of opportunity for dementia prevention could be defined based on MRI measures. For example, the recent World Wide FINGERS (WW-FINGERS) initiative is currently developing the first global network of dementia prevention trials based on the FINGER model [43]. Results from the FINGER MRI exploratory sub-study provide a first reference frame for incorporating MRI outcomes into such large-scale trial networks and offer a hypothesis that can be confirmed or refuted in future trials.

## Data Availability

The datasets generated and/or analyzed during the current study are not publicly available due to the ethics rules and legislation in Finland. For more information, please contact Dr. Tiia Ngandu (tiia.ngandu@thl.fi).

## Abbreviations

AD: Alzheimer’s disease
FINGER: Finnish Geriatric Intervention Study to Prevent Cognitive Impairment and Disability
GM: Gray matter
MRI: Magnetic resonance imaging
NTB: Neuropsychological test battery
TIV: Total intracranial volume
WML: White matter lesions
